# Real-world retrospective study with upadacitinib in patients with severe alopecia areata: A series of 23 cases

**DOI:** 10.1016/j.jdcr.2026.06.020

**Published:** 2026-06-16

**Authors:** Sergio López-Alcázar, Andrés Vidal-González, Marta Feito-Rodríguez, Raúl De Lucas-Laguna, Rocío Maseda-Pedrero, Guillermo Servera-Negre, Elena Sendagorta-Cudós, Natalia Hernández-Cano, Pedro Herranz-Pinto, Rocío Gil-Redondo

**Affiliations:** Department of Dermatology, Hospital Universitario La Paz, Madrid, Spain

**Keywords:** alopecia areata, Janus kinase inhibitors, real-world evidence, severe alopecia areata, treatment outcomes, upadacitinib

## Introduction

Alopecia areata (AA) is an autoimmune non-scarring alopecia with a major impact on quality of life. Janus kinase (JAK) inhibitors have emerged as an effective therapeutic option for severe disease.^1^ Upadacitinib, a selective JAK1 inhibitor approved for several immune-mediated diseases, has shown encouraging results in recent case series and retrospective studies, and recently, phase 3 randomized placebo-controlled trials in moderate-to-severe AA have been completed with positive results, and regulatory approval for this indication is currently under evaluation.^2^ Herein, we report a real-world series of 23 patients with severe AA treated with upadacitinib at a tertiary hospital in Spain.

## Materials and methods

We conducted a retrospective single-center observational study in the Dermatology Department of Hospital Universitario La Paz (Madrid, Spain). We included all consecutive patients with AA who received upadacitinib at our center from the initiation of its use for AA until September 2025. Scalp disease severity for treatment-response analyses was defined according to baseline Severity of Alopecia Tool (SALT) score at treatment initiation, with severe involvement considered as SALT ≥50. Patients with lower baseline SALT scores were included if they had clinically severe disease due to eyebrow, eyelash, nail, or body hair involvement, cosmetically unacceptable scalp hair loss, or relevant inflammatory comorbidities. Upadacitinib was administered at 15 or 30 mg once daily based on age, weight, and disease severity, at the discretion of the treating physician, with dose adjustments allowed according to response and tolerability. Concomitant topical therapies were permitted and used in most patients, whereas no systemic treatments were administered. Medical records were reviewed to collect demographic and clinical data, comorbidities, prior treatments including previous JAK inhibitor exposure, treatment duration and dose, SALT evolution, response in other body areas, adverse events, and patient-reported satisfaction. The study followed the Declaration of Helsinki, and all patients provided written informed consent.

## Results

### Demographic variables, family history, and comorbidities

The cohort included 23 patients with AA. Baseline demographic and clinical characteristics are summarized in Table I. Both pediatric and adult patients were included, including children younger than 12 years, with most patients being adults and a slight female predominance. Mean age at disease onset was during adolescence, and disease duration was long, reflecting a chronic and treatment-refractory population.

**Table I:** Baseline demographic and clinical characteristics of the study population

	*N*
Total *N*	23
Demographic variables	
Sex	
Men	10 (43.5%)
Women	13 (56.5%)
Mean age	28.1 (SD 15.5)
Age range	7-53 y
>18 y	14 (61%)
12-18 y	6 (26%)
<18 y	3 (13%)
Age at disease onset (y)	15.4 (1-44; SD 13.9)
Mean duration of current episode (y)	7.3 (0.2-44; SD 11.9)
Baseline disease severity	
Mean baseline SALT score	52.3 (0-100; SD 35.9)
Baseline SALT category	
SALT 95-100	5 (21.7%)
SALT 50-94	8 (34.7%)
SALT 20-49	5 (21.7%)
SALT 1-19	2 (8.7%)
SALT 0	3 (13%)
Extra-scalp involvement	
Eyebrows	14 (60.9%)
Eyelashes	12 (52.2%)
Beard	5 (21.7%)
Body hair	10 (43.5%)
Nails	3 (13%)
Family history of AA	3 (13%)
Comorbidities	
Any comorbidity	13 (56.5%)
Atopic dermatitis	6 (26%)
Psoriasis/psoriasic arthritis	2 (8.7%)
Psoriasiform dermatitis	1 (4.3%)
Twenty-nails distrophy	1 (4.3%)
Male androgenetic alopecia	1 (4.3%)
Turner syndrome	1 (4.3%)
Autoinmune hypothyroidism	1 (4.3%)
Prior treatments	
Prior JAKi exposure	19 (82.6%)
Tofacitinib	2 (8.7%)
Baricitinib	9 (39.1%)
Tofacitinib > baricitinib	8 (34.7%)
Mean duration of prior JAKi therapy (mo)	29.8 (3-76; SD 21.7)
Mean washout period before upadacitinib (d)	7.9 (0-150; SD 33.4)
Reasons for switching to upadacitinib from other JAKi	
Primary failure	3 (13%)
Secondary failure	7 (30.4%)
Insufficient efficacy	5 (21.7%)
Poor control of comorbidities	2 (8.7%)
Poor control of comorbidities and insufficient efficacy	2 (8.7%)

*N*, Number of patients.

Only 13% of patients (*n* = 3) had a family history of AA. More than half of patients had at least 1 associated comorbidity, most frequently atopic dermatitis and psoriatic disease. Overall, this profile highlights the frequent coexistence of AA with other inflammatory and immune-mediated conditions.

A detailed summary of demographic and clinical characteristics is provided in Table I.

### Previous treatments

Most patients had received multiple prior treatments, including topical, intralesional, and systemic therapies, reflecting a refractory population.

Prior exposure to JAK inhibitors was common, as detailed in Table I. Only 4 patients (17.4%) started upadacitinib without receiving another JAK inhibitor previously. Of these 4 patients, 3 started upadacitinib directly taking advantage of the indication for their comorbidity (atopic dermatitis, *n* = 1; psoriatic arthritis, *n* = 2). The remaining patient had acquired twenty-nail dystrophy, initially treated as psoriasis, who subsequently developed alopecia areata patches on body hair; therefore, upadacitinib was started to control both conditions.

### Reasons for switching to upadacitinib

Among the 19 patients previously exposed to JAK inhibitors, reasons for switching to upadacitinib were heterogeneous and are summarized in Table I. Primary failure (SALT >20 after treatment) to the previous JAK inhibitor was observed in some patients, whereas secondary failure (relapse with SALT >20) was the most frequent cause. In several cases, switching was due to efficacy considered insufficient or inadequate stabilization of the response; we defined these as patients who had achieved SALT <20 but whose real-world cosmetic outcome was not acceptable. In other patients, the main reason for switching was lack of response of comorbidities such as psoriasis or atopic dermatitis, and in some cases the decision was driven by the combination of comorbidity and insufficient efficacy of prior treatment.

### Baseline SALT prior to initiation of upadacitinib

Baseline scalp involvement was variable, with most patients presenting moderate-to-severe disease. Three patients initiated treatment with baseline SALT 0 due to significant extra-scalp involvement or management of inflammatory comorbidities.

In addition, the mean duration of the current episode in our cohort was 7.3 years.

### Involvement in other locations before treatment

Extra-scalp involvement was frequent, particularly affecting eyebrows and eyelashes, as detailed in Table I.

### Dose and duration of treatment with upadacitinib

The mean duration of treatment with upadacitinib was 52 weeks (range 16-96; SD 22). Regarding mean daily dose used in the overall cohort, 60.9% of patients (*n* = 14) received 30 mg/day, 30.4% (*n* = 7) received 15 mg/day, and 8.7% (*n* = 2) received a mean dose of 22.5 mg/day.

When analyzing dose by age, in the adult group (>18 years, *n* = 14), 12 patients were treated with 30 mg/day and 2 with 15 mg/day. Among adolescents aged 12 to 18 years (*n* = 6), 2 received 30 mg/day, 2 received 15 mg/day and 2 underwent a progressive adjustment from 15 to 30 mg/day. In children younger than 12 years (*n* = 3), all were treated with 15 mg/day.

### SALT score evolution during treatment

**Fig 1:**
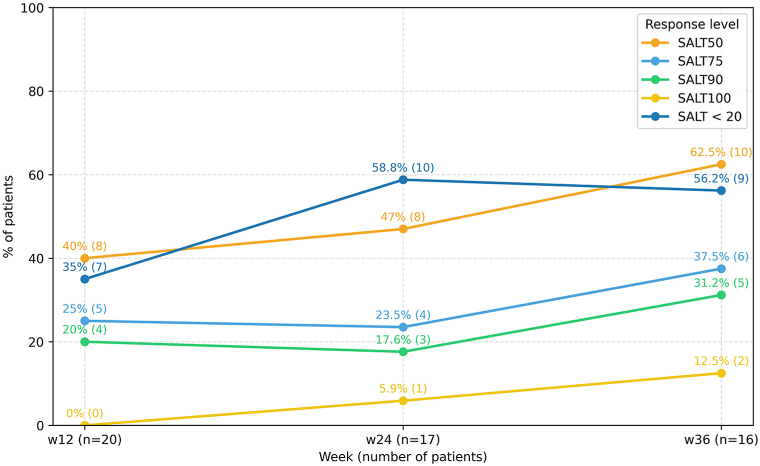
Evolution of the SALT score at weeks 12, 24, and 36.

Treatment outcomes were assessed using both absolute SALT thresholds and relative SALT reduction (percentage change from baseline), the latter inherently reflecting only patients with baseline scalp involvement and therefore not influenced by individuals starting from SALT 0. Serial follow-up of the SALT index at weeks 12, 24 and 36 showed progressive improvement in most patients. At week 12 (*n* = 20), 35% (*n* = 7) had achieved SALT <20, whereas at week 24 (*n* = 17) this proportion increased to 58.8% (*n* = 10) and at week 36 (*n* = 16) it decreased to 56.2% (*n* = 9). This decrease at week 36 is explained by the reduction in sample size over time, as not all patients had reached weeks 24 and 36 of treatment. In terms of SALT50 response, 40% (*n* = 8) had achieved it at week 12, increasing to 47% (*n* = 8) and 62.5% (*n* = 10) at weeks 24 and 36, respectively. In parallel, response rates for SALT75, SALT90 and SALT100 also increased steadily over follow-up. The evolution of response rates over the follow-up period is shown in Fig 1.

In addition, it should be noted that 1 patient who had not achieved a SALT50 response at week 36 achieved complete hair regrowth by week 56. Therefore, although uncommon, late responders to upadacitinib treatment may be observed.

In the overall analysis at the time of data collection (September 2025), 16 of 23 patients (69.5%) had SALT <20 (56.5% excluding the 3 patients with baseline SALT 0). In addition, 13 patients (56.5%) achieved SALT50, 10 (43,5%) achieved SALT75, 7 (30.4%) achieved SALT90 and 3 (13%) had complete scalp regrowth (SALT100). Three patients who started treatment with SALT 0 maintained complete regrowth throughout follow-up. Final mean SALT was 24.4 (SD 33.8), with a reduction of 45.4% and 27.8 points (SD 45.4 and 30.5, respectively); this increased to a reduction of 52.2% and 32 points (SD 44.9 and 30.6, respectively) if we exclude patients who started from SALT 0. Additionally, all patients with baseline SALT 0 maintained complete scalp regrowth throughout follow-up. Table II summarizes the overall results and outcomes stratified by baseline severity groups in terms of relative SALT response and absolute SALT ≤20.

**Table II:** Treatment response according to baseline SALT severity

	SALT <20	SALT50	SALT75	SALT90	SALT100
SALT 95-100 (*n* = 5)	1 (20%)	1 (20%)	1 (20%)	1 (20%)	0
SALT 50-94 (*n* = 8)	6 (75%)	6 (75%)	6 (75%)	4 (50%)	1 (12.5%)
SALT 20-49 (*n* = 5)	4 (80%)	4 (80%)	2 (40%)	2 (40%)	2 (40%)
SALT 1-19 (*n* = 2)	2 (100%)	2 (100%)	1 (50%)	0	0
SALT 0 (*n* = 3)	3 (100%)	-	-	-	-
Total *N* (*n* = 23)	16 (69.5%)	13 (56.5%)	10 (40.5%)	7 (30.4%)	3 (13%)

### Response in other body locations

A total or near-total regrowth was achieved by 71.4% (*n* = 10) of patients with eyebrow involment, whereas 28.6% (*n* = 4) had partial regrowth. For eyelashes, total or near-total regrowth was observed in 66.6% (*n* = 8), partial regrowth in 25% (*n* = 3) and no response in 8.3% (*n* = 1). Among patients with beard involvement, 40% (*n* = 2) showed total or near-total regrowth, whereas 60% (*n* = 3) experienced partial regrowth. For body hair, 70% of affected patients (*n* = 7) showed total or near-total improvement, while the remaining 30% (*n* = 3) experienced partial improvement. Regarding nail involvement, 33.3% (*n* = 1) of patients showed total or near-total improvement, whereas the remaining 66.6% (*n* = 2) experienced partial improvement.

### Reasons for treatment discontinuation

At the time of analysis, 78.3% of patients (*n* = 18) continued treatment with upadacitinib. Only 21.7% of patients (*n* = 5) had discontinued treatment for different reasons: primary failure (*n* = 1), secondary failure (*n* = 1), comorbidity (atopic dermatitis) associated with low efficacy (*n* = 2), and poor control of psoriatic comorbidity (*n* = 1).

### Safety

Regarding safety, 39.1% of patients (*n* = 9) experienced an adverse event during follow-up, all of mild to moderate intensity. Acneiform reactions were the most common adverse event, observed in 5 patients. In addition, we recorded: mild psoriasiform reaction (*n* = 1), plantar warts (*n* = 1), molluscum contagiosum (*n* = 1), bacterial pneumonia (*n* = 1) and hypercholesterolemia (*n* = 1). None of these adverse events required permanent discontinuation of treatment or relevant dose adjustments, suggesting an acceptable safety profile in this cohort.

### Patients’ perception and satisfaction

Patients’ subjective perception of improvement with upadacitinib was mostly positive. Regarding treatment with prior JAK inhibitors (*n* = 16), 37.2% of patients (*n* = 6) reported feeling much better and 31.2% (*n* = 5) reported feeling better, while the remaining 31.2% (*n* = 5) perceived no significant change. In terms of overall satisfaction (*n* = 20), 55% of patients (*n* = 11) stated they were very satisfied with treatment and 30% (*n* = 6) were satisfied, while 35% (*n* = 7) considered themselves indifferent.

## Discussion

Our results are consistent with other real-world series and retrospective studies showing high efficacy of upadacitinib in severe AA, including patients previously treated with other JAK inhibitors. In our cohort, nearly 70% of patients achieved SALT <20 and 13% achieved SALT100, with response rates comparable to those previously reported. The lower mean baseline SALT score may be explained by partial responses to prior treatments, treatment switching driven by comorbidities, and inclusion of 1 patient with exclusive extracranial involvement.

In addition, when interpreting our results, we must consider the long mean disease duration in our cohort, as several studies indicate that an episode duration longer than 4 years may predict a poor response to JAK inhibitors.^3^

As a strength of our study, it should be highlighted that our series reports one of the longest treatment durations with upadacitinib in AA among the series published to date. Table III compares the findings of our study with those of previously published series.

**Table III:** Comparison of our study with other published series

	Baseline SALT	Prior JAKi use	Upadacitinib dose	Treatment duration	Final SALT	Non-responders	Adverse events
Picone et al^4^ (*n* = 15)	Mean: 93.1	No	15 mg	40 wk	SALT50: 60% (9) SALT75: 40% (6) SALT90: 26.6% (4)	6	Elevated CPK Acne URTI
Johnston et al^5^ (*n* = 3)	Mean: 86-90	No	30 mg	3-8 mo	SALT100: 100% (3)	0	-
Chiricozzi et al^6^ (*n* = 19)	Mean: 95.1	No	30 mg (2 reduced to 15 mg)	40 wk	Mean SALT: 39.2	8	-
Wang et al^7^ (*n* = 17)	Moderate: 4 Severe: 10 Very severe: 3	Abrocitinib: 4 Baricitinib: 6	15 mg	24 wk	SALT50: 88.2% (15) SALT75: 58.8% (10) SALT90: 35.3% (6)	2	Folliculitis
Chambres et al^8^ (*n* = 22)	Mean: 85	Baricitinib: 9	30 mg Except: −1 45 mg (Crohn disease) −1 15 mg (pediatric)	Media: 9.8 wk	SALT >20: 23% (5) 0 < SALT <20: 9% (2) SALT 0: 68% (15)	0	Acne Asthenia URTI Nausea Elevated GGT Cellulitis
Li et al^9^ (*n* = 23)	Mean: 73.9	Abrocitinib: 2 Baricitinib: 9 Tofacitinib: 5	7.5-30 mg	36 wk	Mean SALT reduction: 67.3 SALT50: 100% (23) SALT100: 63.6% (15)	0	Acne Folliculitis URTI COVID-19
López-Alcázar et al (*n* = 23)	Mean: 52.3	Tofacitinib: 2 Tofa > Baricitinib: 8 Baritinib: 9	30 mg: 14 15 mg: 7 30 mg > 15 mg: 2	Mean: 52 wk	Mean SALT reduction: 52.2% SALT <20: 69.5% (16) SALT50: 52.1% (12) SALT75: 39.1% (9) SALT90: 30.4% (7) SALT100: 13% (3)	5	Acne Psoriasiform reaction Plantar warts Molluscum contagiosum Pneumonia Hypercholesterolemia

*URTI*, Under respiratory tract infections.

When analyzing prognostic factors for AA response to upadacitinib in our series, younger patients (<35 years), with non-early onset of AA (beyond early childhood) and with a relatively short disease duration, especially less than 8 years, showed better hair regrowth with upadacitinib. Sex did not appear to meaningfully influence response, nor did the presence of atopic or psoriatic comorbidities, as patients with these conditions achieved SALT reductions comparable to the rest of the cohort. Regarding prior treatment with JAK inhibitors, the 3 JAK inhibitor-naïve patients in our cohort showed more favorable responses; nevertheless, clinically relevant regrowth was also observed in patients previously treated with other JAK inhibitors, especially when the reason for discontinuation had been secondary loss of efficacy rather than primary non-response, suggesting that switching to upadacitinib may be a useful strategy to recapture response in selected cases. However, these findings should be interpreted with caution given the limited sample size (only 23 patients), and larger, prospective studies are needed to confirm them.

The main limitations of our study include its observational, retrospective, single-center design, the relatively small sample size, and the fact that around 30% of patients had not yet reached week 36 of follow-up at the time of analysis. However, the real-world nature of the cohort and the presence of complex patients with multiple comorbidities and prior treatments, including JAK inhibitors, add value to the interpretation of the data.

## Conclusions

In conclusion, in this series of 23 patients with severe AA, upadacitinib showed significant clinical efficacy, with a marked reduction in SALT in most patients and complete scalp regrowth in a non-negligible proportion. Response in other locations such as eyebrows, eyelashes, nails and body hair was also relevant, translating into high subjective satisfaction in a substantial proportion of the cohort. The safety profile was favorable, with mild to moderate adverse events that did not require treatment discontinuation.

Upadacitinib is thus positioned as an effective and safe therapeutic option for patients with severe AA, even in the context of refractoriness to other JAK inhibitors; however, prospective studies and controlled clinical trials with larger sample sizes and longer follow-up will be necessary to confirm and expand these findings.

## Conflicts of interest

None disclosed.
